# Pulmonary Tuberculosis and *Mycobacterium bovis,* Uganda

**DOI:** 10.3201/eid1501.080487

**Published:** 2009-01

**Authors:** Frederick Byarugaba, Eric Marcel Charles Etter, Sylvain Godreuil, Patrice Grimaud

**Affiliations:** Mbarara University of Science and Technology, Mbarara, Uganda (F. Byarugaba); Centre de Cooperation Internationale en Recherche Agronomique pour le Développement, Montpellier, France (E.M.C. Etter); Hôpital Arnaud-de-Villeneuve, Montpellier (S. Godreuil); Centre de Cooperation Internationale en Recherche Agronomique pour le Développement, N’Djamena, Chad (P. Grimaud)

**Keywords:** Mycobacterium bovis, tuberculosis, Uganda, letter

**To the Editor:** In 2005, prevalence of human tuberculosis (TB) in Uganda was 559 cases/100,000 population (*1*). In 2002, the average number of extrapulmonary TB cases in humans, considered a crude indicator of the level of bovine TB, was 7.5% of TB cases for Uganda and 6% for Mbarara district, the main Ugandan milk basin (*2*). Worldwide, the proportion of human cases caused by *Mycobacterium bovis* has accounted for 3.1% of all forms of TB (*3*). Although zoonotic TB is more often reported as an extrapulmonary disease, recent publications report that 0.4%–10% of sputum isolates from patients in African countries are *M. bovis* (*3*). These studies, however, give little information about the cattle environment. A 2002 survey of dairy cattle in Mbarara district reported that 74% of herds and 6% of individual animals were reactive to the single tuberculin test (*4*). However, this test does not differentiate between *Mycobacterium* species involved. We therefore explored whether *M. bovis* might be a major threat to human health in this region.

From September 2004 through January 2005, we surveyed 658 patients who had been admitted to the Mbarara University Teaching Hospital TB ward after positive bacterial findings for at least 3 sputum smears or positive chest radiographs for smear-negative patients. Of 90 randomly selected patients, only 70 samples were available for analysis to differentiate the species in the *M. tuberculosis* complex; the other samples were excluded because of contamination, lack of mycobacteria growth on culture, or postal delay in transportation of sample. The questionnaire asked about patients’ demographic data (including occupation), association with cattle, and milk consumption habits. Genomic DNA was extracted from the pellet culture of Middlebrook 7H9 broth (Difco; Cergy, France) as described previously (*5*). DNA samples were used to carry out PCRs and hybridization processes; we used the GenoType MTBC kit (Hain Lifescience GmbH; Nehren, Germany) for differentiation in the *M. tuberculosis* complex, especially between *M. tuberculosis* and *M. bovis* species (*6*).

Questionnaire responses showed that 27/64 (42.2%; 6 did not answer) patients had a history of raw milk consumption; nevertheless, 20/24 (83%; 3 did not answer) reported that they boiled fresh milk before consuming it, as did 54/60 (90% of all patients; 10 did not answer). Eating undercooked or raw meat was reported by 91% of the patients. Most patients were adult males (ratio 2.14:0.97 male:female for the district population); 8.6% were <18 years old (56% in the district); and average number of persons in household was 5.7 vs. 4.8 for the district (*7*). Of the samples, 8.6% were from extrapulmonary sites.

After amplification and hybridization of sample DNA, 69 samples were found to be *M. tuberculosis,* and 1 was not a *Mycobacterium* species. Our sampling method would detect at least 1 case of *M. bovis* in *n* patient specimens if the prevalence of bovine TB was > *p*(0.033%) according to the formula in which *a* is the first order error (5%):


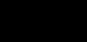


 Because of the change in sample size, the limit prevalence was redetermined by using the inverse of the formula above:


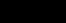


If at least 1 sample was positive for *M. bovis,* the prevalence of bovine TB among patients would be >4.2%. However, the prevalence of *M. bovis* was <4.2% and confirmed the low-level involvement of *M. bovis* in human TB in Mbarara district. These findings are consistent with previous work in Uganda’s capital, Kampala, and in other African or Asian countries (*2*,*8*,*9*). The estimation of extrapulmonary cases among all TB cases (95% confidence interval 2%–15.2%) did not differ from the official estimate. We can add, using the second formula shown above, that among the 6 extrapulmonary TB cases, the prevalence of *M. bovis* is <39.3%. Our results come from a population in a highly rural area (91.5% of the population in Mbarara district) (*7*), where the high prevalence of animal TB has been reported.

These results could be explained by the patients’ consumption habits, which reduce the risk for contamination. Even if bovine TB could also be found in other farm or wild animals, it seems to have a minor effect on public health. Zoonotic TB appeared to not be a major public health problem in Mbarara district. However, this finding could also result from underdiagnosis of extrapulmonary TB, from prevalence of *M. tuberculosis* being so high that in proportion *M. bovis* is a minor problem, or from rural populations’ difficult access to TB diagnosis (directly observed therapy case detection rate in Uganda in 2005 was 37%) (*1*).
